# Virtual connection between older people with dementia and families during COVID-19 pandemic

**DOI:** 10.1093/geroni/igaa057.3514

**Published:** 2020-12-16

**Authors:** Lillian Hung

**Affiliations:** University of British Columbia, Vancouver, British Columbia, Canada

## Abstract

People staying in hospitals need more support to cope with the lockdown and visitor restriction during the COVID-19 pandemic, especially for older people with cognitive or physical impairment. Everyday technology such as a touchscreen tablet has great potential to support person-centred care. We aimed to support the adoption of tablets for hospitalized people with dementia to connect with families and friends. A patient-oriented research approach was employed to co-produce the toolkit. We are a transdisciplinary team, including a medical student, physicians, nurses, patients, and family partners. We facilitated staff focus groups (n=3), and conducted stakeholders’ interviews (n=4) to gain a more comprehensive understanding of users’ needs. The sample included ten patients, ten family members, 40 staff members, nurses, care workers, physicians, and unit clerks (n=40). The Consolidated Framework for Implementation Research (CFIR) guided the research design and qualitative analysis. A toolkit was developed based on participants’ perspectives on what needs to be in place to support successful adoption. We developed a mobile tablet with one mechanical arm and one leg on wheels. Participants reported impacts: (a) it puts a smile on the patient’s face, (b) it alleviates anxiety and worries on both sides, and (c) it reduces responsive behaviors. The conceptual framework CFIR provides helpful guidance in identifying barriers to implementation. Working with users including patient and family partners to explore possible solutions was key to our success. Future research should engage patient and family partners to seek proactive strategies to address obstacles to advance the science of technology implementation.

